# Comprehensive review of *Plasmodiophora brassicae*: pathogenesis, pathotype diversity, and integrated control methods

**DOI:** 10.3389/fmicb.2025.1531393

**Published:** 2025-02-06

**Authors:** Xueliang Xu, Caiyun Wu, Fan Zhang, Jian Yao, Linjuan Fan, Zirong Liu, Yingjuan Yao

**Affiliations:** Jiangxi Provincial Key Laboratory of Agricultural Non-Point Source Pollution Control and Waste Comprehensive Utilization, Institute of Agricultural Applied Microbiology, Jiangxi Academy of Agricultural Sciences, Nanchang, China

**Keywords:** clubroot disease, *Plasmodiophora brassicae*, infection process, effectors, physiological pathotypes, management strategies

## Abstract

Clubroot disease is an important disease of cruciferous crops worldwide caused by *Plasmodiophora brassicae*. The pathogen *P. brassicae* can infect almost all cruciferous crops, resulting in a reduction in yield and quality of the host plant. The first part of this review outlines the process of *P. brassicae* infestation, effectors, physiological pathotypes and identification systems. The latter part highlights and summarizes the various current control measures and research progress on clubroot. Finally, we propose a strategic concept for the sustainable management of clubroot. In conclusion, this paper will help to deepen the knowledge of *P. brassicae* and the understanding of integrated control measures for clubroot, and to lay a solid foundation for the sustainable management of clubroot.

## 1 Introduction

Clubroot disease caused by obligate biotrophic, parasitic protist *Plasmodiophora brassicae* Woronin is a major constraint in the current production of cruciferous crops (Ahmed et al., 2020; Hwang et al., 2012; Saharan et al., 2021). The host range of *P. brassicae* is wide, and all 330 genera and 3,700 species of the Brassicaceae family may be hosts of *P. brassicae*, including rape, Chinese cabbage, bok choy, kale, radish, cauliflower, mustard and other cultivated and wild species (Chai et al., 2014; Hwang et al., 2012). The infestation of *P. brassicae* in the host leads to the slow growth of tumors on the roots, which hinders the plant’s capacity to absorp water and nutrients. This may ultimately limit the host’s growth and, in severe situations, lead to death (Howard et al., 2010). It has been estimated that *P. brassicae* causes 10–15% yield loss in cruciferou crop production globally each year (Dixon, 2009a; Strehlow et al., 2014). In addition, the infection of *P. brassicae* also has an adverse effect on the quality of these crops (Howard et al., 2010). The spread of *P. brassicae* is extremely fast, and its geographical distribution is constantly expanding rapidly. It is reported that *P. brassicae* is widely distributed in more than 80 countries such as Europe, North America, Latin America, Oceania and Asia, causing economic losses of hundreds of millions every year (Javed et al., 2023).

**Figure 1:**
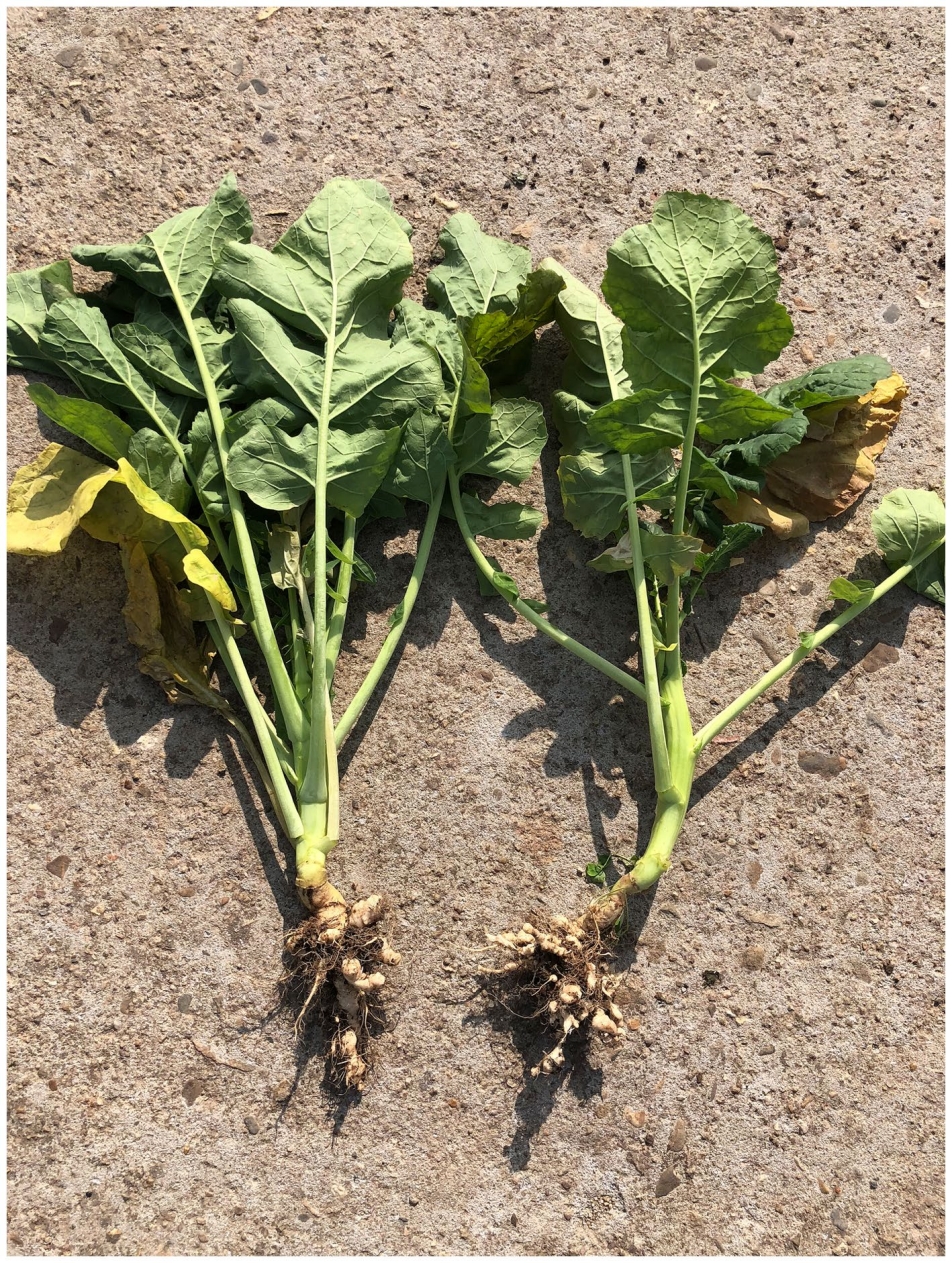
Symptoms of clubroot in *Brassica napus* infected by *Plasmodiophora brassicae.*

The *P. brassicae* can infest both seedling and adult stages of the host plant causing damage. The absorption of water and nutrients in the infected plant is blocked, affecting the normal growth and development of the plant. In the early stage of infection, the aboveground part of the plant has no obvious symptoms, and some plants grow slowly. In the middle stage of infection, the plant is dwarfed and some leaves turn yellow and wither. In the late stage of infection, root galls of varying sizes, shapes and locations form on the roots (Figure 1), leading to plant death in severe cases (Greer et al., 2023; Zamani-Noor et al., 2022).

## 2 Process of infestation by *Plasmodiophora brassicae*

The lifecycle of *P. brassicae* consists of three phases, survival in soil, primary root hair infection, and secondary cortical infection (Feng et al., 2014; Ludwig-Müller et al., 2009). The resting spores are the main body of the soil survival stage and the initial stage of the life cycle of *P. brassicae*. Under appropriate conditions, resting spores of *P. brassicae* in the soil germinate and release primary zoospores with biflagellates, which subsequently move to the vicinity of the host plant roots to begin the process of infestation (Olszak et al., 2019; Schuller and Ludwig-Müller, 2016).

The process of infection of host plants by the *P. brassicae* has now been more clearly described (Ji et al., 2014; Kageyama and Asano, 2009). The infection of *P. brassicae* to host roots is generally divided into two stages. The first stage is the primary root hair infection, which lasts about 7 days. Around 1 day post infection (dpi), primary spores puncture the host cell wall and enter root hair cells or epidermal cells, producing uinucleate primary plasmodium. At 1–3 dpi, the uinucleate primary plasmodium develop into multinucleated zoosporangial plasmodium through mitosis. Then, at 3–4 dpi, large numbers of uninucleate secondary spores are produced with cytoplasmic cleavage. At 4–7 dpi, the uninucleate secondary zoospores were released into the living epidermal cells of the root hair cells, and finally two mononuclear secondary spores were combined in the epidermal cells of the roots to form a diploid uninucleate zygote. The second stage is secondary cortical infection, which takes about 17 days. At 8 dpi, uninucleate secondary plasmodium appeared in the cortical cells of the host, indicating the beginning of secondary infection. Next, at the 10 dpi, the uninucleate secondary plasmodium developed into binucleate secondary plasmodium. This then continues to develop into tetranucleate and multinucleate secondary plasmodium resulting in root expansion. The uninucleate resting spores are produced on day 24 and are released into the soil after maturation to complete a life cycle and enter the next infection cycle (Liu et al., 2020a; Liu et al., 2020b).

## 3 Effectors of *Plasmodiophora brassicae*

Plant defense against pathogens relies primarily on their own immune system, which recognizes the pathogens and initiates an effective defense response. Conversely, the key to successful infestation of a plant host by a pathogen lies in the manipulation of the host’s defense system through effectors (Cui et al., 2015; Dodds and Rathjen, 2010; Dodds et al., 2024). The identification of effectors is a prerequisite for understanding how pathogens manipulate immune responses and disease resistance genes in plants (Pérez-López et al., 2018). However, *P. brassicae* is an obligate biotroph cannot be cultured away from its host, representing a major challenge in the research of their effectors. With the rapid development of genomic, transcriptomic, proteomic and other biotechnologies, particularly the powerful predictive capabilities of bioinformatics tools, an increasing number of effectors from *P. brassicae* have been identified and predicted (Schwelm and Ludwig-Müller, 2021; Siemens et al., 2009). The identified and predicted putative effectors of *P. brassicae* are summarized in Table 1.

**Table 1:** List of putative effectors *Plasmodiophora brassicae.*

Effector	Accession number	Putative annotated function	Expression peaks	Applied technologies or omics methods	References
PbBSMT	AFK13134	Benzoic acid/salicylic acid methyltransferase	Three and 4 weeks after infection	Genome sequencing	Ludwig-Müller et al. (2015) and Rolfe et al. (2016)
PbGH3	–^a^	Modify auxin and jasmonic acid	–	Genome sequencing, transcriptome sequencing and filtering, conjugate synthetase test,	Schwelm et al. (2015)
PbCYP3 (PBRA_003184)	–	Restored fungal colonization in plant tissues	Germinating spores, plasmodia and maturing spores,	Prediction of immunophilins, domain analysis, subcellular localization, transcriptome analysis,	Singh et al. (2018)
PBCN_002550	QGW67292	Induced cell death	5 and 7 days postinoculation	Transcriptome analysis; yeast signal sequencetrap assay; agrobacterium-mediated plant virus transient expres-sion in Nicotiana benthamiana leaves	Chen et al. (2019)
PBCN_005499	QGW67305	Induced cell death	5 and 7 days postinoculation	Transcriptome analysis; yeast signal sequencetrap assay; agrobacterium-mediated plant virus transient expres-sion in Nicotiana benthamiana leaves	Chen et al. (2019)
PbRING1	PBRA_000499	E3 ubiquitin ligase	plasmodia stage	Yeast invertase assay, heterologous expression, determination of the E3 ligase activity	Yu et al. (2019)
SSPbP22 (PBRA_008980)	CEP02396	Kinase	14, 21 and 28 days postinoculation	RNA-Seq analysis, Signal peptide validation assay, Kinase activity assay, Dot-blot analysis, Protein localization	Pérez-López et al. (2020)
SSPbP53 (PBRA_008207)	CEP00895	Cysteine protease inhibitor	21 and 28 days postinoculation	RNA-Seq analysis, genome-wide transcriptomic analysis, Western blot analysis, activity-based protein profiling assay	Pérez-López et al. (2020) and Pérez-López et al. (2021)
Indole-3-acetaldehyde dehydrogenase	SPQ96145.1	Involvement in indole acetic acid synthesis	21 days after inoculation	Bioinformatics pipeline, protein modeling and orthologous comparisons with effectors from other pathosystems	Galindo-González et al. (2021)
PBZF1	QGW67289	RxLR effector	Resting spores	Yeast two-hybrid assay, glutathione S-transferase pull-down assay, and bimolecular fluorescence complementation assay, sequence polymorphism analysis,	Chen et al. (2021)
PbPE13	CDSF01000098.1	Enhanced *Phytophthora infestans* Infestin 1-induced programmed cell death response	28 days postinoculation	Yeast signal sequence trap assay, Subcellular localization, Agrobacterium co-infiltration, Cell death assay	Hossain et al. (2021)
Pb4_102097	–	Induced cell death and produce H_2_O_2_ accumulation	21 days postinoculation	Agroinfiltration-mediated transient expression in *Nicothiana benthamiana*, PacBio technic	Zhan et al. (2022)
Pb4_108104	–	Induced cell death and produce H_2_O_2_ accumulation	21 days postinoculation	Agroinfiltration-mediated transient expression in *Nicothiana benthamiana*, PacBio technic	Zhan et al. (2022)
PbChiB2	CEP01301	Inhibit chitin-triggered immunity	Maturing spores	Signal peptide validation assay, western blot, carbohydrate sedimentation assay	Muirhead and Pérez-López (2022)
PbChiB4	CEP03198	Inhibit chitin-triggered immunity	Germinating spores	Signal peptide validation assay, western blot, carbohydrate sedimentation assay	Muirhead and Pérez-López (2022)
PbHMWSP34	–	Inhibit the expression of jasmonic acid, ethylene, and several salicylic acids signaling pathway marker genes	–	Yeast signal sequence trap assay, reactive oxidative species burst assay, transcriptome analysis	Feng et al. (2024)
PbZFE1	LC773734	Transcription factor-type effector	40 days postinoculation	Yeast one-hybrid screening, northern blot analysis, southern blot analysis, western blot analysis, electrophoretic mobility shift assay	Ando et al. (2024)
PbE3-2	ON394061	Really Interesting New Gene-type E3 ubiquitin ligase, suppresses plant immune response	–	Yeast invertase secretion assay, apoplastic fluid assay, ubiquitination assay, split-LUC complementation assay	Li C. et al. (2024)
Pb257	CDSF01000 001.1	Induced cell death and induced root enlargement	4 days postinoculation	Yeast signal sequence trap assay, virus‑induced gene silencing assay, transient over‑expression, oxygen burst detection	Yang et al. (2024)

^aDashes indicate unknown.^

PbBSMT was reported to be the first effector of *P. brassicae* to be identified. It is a protein with homologies to plant SABATH-type methyltransferases that reduces or removes the host salicylic acid (SA) defense signaling pathway by methylating SA (Ludwig-Müller et al., 2015; Rolfe et al., 2016). Furthermore, transcriptome analyses revealed that among the genes, *PbBSMT* was one of the most highly expressed during *P. brassicae* infestation, suggesting that it plays an important role in the infestation process (Ciaghi et al., 2019). Chen et al. (2019) found that 28 secreted proteins expressed during the primary infection stage of *P. brassicae* infestation inhibited cell death to facilitate their propagation and colonization. The response mechanism of resistant and susceptible *Brassica napus* to *P. brassicae* infestation was assessed through transcriptomic analysis, which identified 21 gene sequences associated with secretory proteins that correlate with the virulence strength of *P. brassicae*. Among them, a gene similar to indole-3-aldehyde dehydrogenase, with ID SPQ96145.1, is involved in the synthesis of indole acetic acid and is a promising candidate effector. (Galindo-González et al., 2021). Yang et al. (2022a, 2022b) found that primary zoospores of *P. brassicae* released many effectors during infestation of *B. rapa* and their expression levels were significantly induced. In addition, 32 small secreted proteins have been reported to be highly expressed during the secondary infestation of *P. brassica*. Among these, SSPbP22 was localized in both the cytoplasm and nucleus and was validated as a kinase (Pérez-López et al., 2020), while SSPbP53 is a papain-like cysteine protease (PLCP) inhibitor that suppresses PLCP activity in six susceptible cruciferous hosts (Pérez-López et al., 2021). Zhan et al. (2022) screened 518 secretory proteins from the *P. brassicae* genome, among which 55 candidate effectors can inhibite cell death induced by Bcl-2-associated X protein, and 21 candidate effectors can inhibit immunity induced by the bacterial pathogen *Pseudomonas syringae* pv. tomato strain DC3000 of *avrRps4* in *Arabidopsis*.

## 4 Physiological pathotypes of *Plasmodiophora brassicae*

Pathogens can rapidly adapt to changing environments, and the generation of pathogen types or races is often achieved through mutations in single effector genes. Horizontal gene transfer (HGT) is very common in protists (Sanders, 2006; Shi-Kunne et al., 2019), and HGT is also a means for pathogens to broaden their host range (Mehrabi et al., 2011; Ochman et al., 2000). Therefore, some scholars have suggested that *P. brassicae* is also a species that can proximally perform horizontal gene transfer (Zhang et al., 2015; Zheng et al., 2018).

Pathogenic differentiation of physiological microspecies exists in *P. brassicae*. The *P. brassicae* isolated from different regional sources may have different genetic backgrounds, resulting in different disease phenotypic characteristics on specific host plants. The *P. brassicae* can be classified into different pathotypes (physiological races) based on their virulence patterns on different hosts plants (Liu et al., 2022). Studies have revealed that the population structure of *P. brassicae* is changing, conferring diversity and heterogeneity to its populations (Hasan et al., 2021). The genetic diversity of *P. brassicae* contributes to the mutation of its field populations into multiple pathotypes, and the large variation in pathogenicity of different pathotypes increase the difficulty of controlling these pathogens (Yang et al., 2020a). The identification of pathogen types is very important. This is due to the fact that without knowledge of the pathogen types and their virulence in the field, it is difficult to breed the appropriate resistance in host cultivars and for growers to choose the best variety or cultivar. Therefore, global efforts have been undertaken to develop accurate and reproducible identification systems or techniques for *P. brassicae* to ensure precise identification and control of different pathotypes (Hollman et al., 2023; Zamani-Noor and Jedryczka, 2024; Zeng et al., 2024).

### 4.1 Identification methods

During the past decades of research on *P. brassicae*, researchers have developed several pathotype classification systems based on serial host sets of *Brassica* species that exhibit resistance or partial resistance to *P. brassicae* in different hosts. The Williams differential system was the earliest system developed and used four hosts (Badger shipper, Jersey Queen, Laurentian and Wilhelmsburger) for identification. Theoretically, this system can distinguish 16 pathotypes or physiological races (Williams, 1966). The host set for European clubroot differential (ECD) was identified by selecting five from each of *B. rapa*, *B. napus* and *B. oleracea*, and pathotypes of *P. brassicae* were coded using a binary notation system. This is currently the universal identification system for clubroot pathogens in Europe (Buczacki et al., 1975). Subsequently, Somé et al. (1996) selected three cultivars of *B. napus* for identification and classified the *P. brassicae* prototypes into five types. Kuginuki et al. (1999) developed a *P. brassicae* pathotype identification system that is more suitable for Japan by using 18 hosts of a Japanese clubroot-resistant (CR) F1 hybrid (F1) cultivars and *Brassica rapa* lines for identification. The Canadian Clubroot Differential (CCD) set selected 13 Brassica hosts for assessment of *P. brassicae* populations, with a total of 17 detectable pathotypes. The system uses a combination of letters and numbers to designate pathotype (Strelkov et al., 2018). Pang et al. (2020) used a set of eight differential inbred lines of Chinese cabbage that contain clubroot-resistant (*CR*) genes as hosts to develop a sinitic clubroot differential (SCD) system. This system was designed to identify hosts of *P. brassicae* in China and is a more suitable system for determining the pathotype of *P. brassicae* in the region.

Although the molecular basis for the classification of *P. brassicae* pathotype is unclear, one viewpoint that is generally shared by most researchers is that differences between pathotypes ultimately reflect differences in their genetic composition, that is, the presence, absence, or differential expression of certain pathogenicity-associated genes (Zhang et al., 2015). Therefore, classification of rhizobial pathotype at the molecular level is a relevant research trend (Kubo et al., 2017). The development of specific molecular markers related to specific pathotypes will serve as a crucial tool for identifying and monitoring *P. brassicae* populations. Previous studies have reported several molecular markers that can be used to identify different pathotypes. Manzanares-Dauleux et al. (2000) identified a specific random amplified polymorphic DNA (RAPD) marker, OPL14_1200_, for pathotype 1 of *P. brassicae* based on the identification system established by Somé et al. (1996) that can be used to rapidly and reliably identify isolates of pathotype 1. The *Cr811* gene can be used as a molecular marker to distinguish between pathotype 5 and other pathotypes based on the Williams differential set identification system (Zhang et al., 2015). Zhou et al. (2018) developed primers P5XF3/P5XR3 and TaqMan probe P5XP3 based on the 18S-ITS region of *P. brassicae* for rapid and reliable diagnosis and quantification of pathotype 5. Zheng et al. (2019) identified six genes unique to pathotype 4 identified by the Williams difference set, *PBRA_003263* and *PBRA_003268* were present in all P4 isolates, *PBRA_000003* and *Novel512* was present in P4-1, and *Novel137 PBRA_005772* was present in P4-2. These genes can be used as molecular markers to distinguish pathotype 4 from other pathotypes and also to distinguish between different types of pathotype 4. Yang et al. (2020a) found three molecular marker genes that screened for pathotype 7. *PBRA_000303* appeared only in pathotype 7 identified based on the Williams difference set; *PBRA_006533* and *PBRA_009559* appeared in pathotype 7 and one genotype of pathotype 4.

Restriction site-associated DNA sequencing (RADseq) employs high-throughput sequencing technology to identify polymorphic genetic markers within genomes. It not only facilitates the rapid and low-cost identification of a large number of polymorphisms within species, but also has fueled studies in ecological, evolutionary and conservation genomics (Davey and Blaxter, 2010; Andrews et al., 2016). Holtz et al. (2018) detected *P. brassicae* in Canada as two distinct and highly divergent populations using RADseq with the removal of the host’s DNA and the endophyte’s DNA. Simple sequence repeats (SSRs) are frequently used as markers for genetic analyses of many eukaryotic organisms (Guichoux et al., 2011). Kubo et al. (2017) developed SSR markers for *P. brassicae* to classify 24 isolates of *P. brassicae* from Japan, and found that there was a close genetic relationship between isolates of pathotype 2 and pathotype 4.

However, these identification method s have certain limitations (Table 2). Traditional identification systems typically require a considerable amount of time, usually around 2 months, making them both time-consuming and labor-intensive (Holtz et al., 2021; Manzanares-Dauleux et al., 2000). Moreover, the pathogenicity of *P. brassicae* spores is influenced not only by environmental factors, such as temperature and pH, but also by their maturity. This interplay can diminish the accuracy and consistency of pathotype identification (Sharma et al., 2011; Tso et al., 2022). Molecular identification methods differ in terms of sensitivity, scalability, accessibility, and operational costs. A major challenge is to identify polymorphisms that provide consistent genomic and phenotypic clustering of pathotypes. Moreover, it is difficult to distinguish individual pathotypes using unique sequences in one region of the genome and may be necessary to rely on multiple regions for identification. In addition, primers designed for molecular identification need to be specific enough to avoid the amplification of non-target genes (Tso et al., 2021).

**Table 2:** Identification methods of pathotypes of *Plasmodiophora brassicae.*

Pathotyping platforms	Identification methods	Advantages	Disadvantages	References
Traditional identification methods	Williams differential system	Global adaptability and minimal effort required	Small detection capacity, time-consuming and labor-intensive	Williams (1966)
	European clubroot differential (ECD) system	Global adaptability and extensive detection capability	High workload, complex naming rules, time-consuming and labor-intensive	Buczacki et al. (1975)
	Somé differential system	Minimal workload and a limited number of hosts	Small detection capacity, geographically limited, time-consuming and labor-intensive	Somé et al. (1996)
	Kuginuki differential system	Extensive detection capability	Heavy workload, geographically limited, time-consuming and labor-intensive	Kuginuki et al. (1999)
	Canadian clubroot differential (CCD) system	Extensive detection capability, global applicability, and simple naming rules	Heavy workload, time-consuming and labor-intensive	Strelkov et al. (2018)
	sinitic clubroot differential (SCD) system	Large detection capability, high detection accuracy and stable detection results	Host acquisition is difficult, time-consuming and labor-intensive	Pang et al. (2020)
Molecular identification methods	Specific molecular markers	Minimal workload, shorter time-consuming and more scalable	Requires specialized instrumentation	a
	Restriction site-associated DNA sequencing (RADseq)	Minimal workload, shorter time-consuming and fewer limitations	Requires specialized instrumentation, higher cost and complex operation	–
	Simple sequence repeats (SSRs)	Minimal workload, shorter time-consuming and simple operation	Requires specialized instrumentation and constrained by limitations	–

^aDashes indicate unknown.^

### 4.2 Single-spore isolation technology

The *P. brassicae* in most studies originated from field populations, i.e., isolated from root galls or plants of field-incurred hosts. However, field populations typically consist of a mixture of different pathotypes, leading to the possibility that pathotypes with lower frequencies may be masked by others that occur at higher frequencies (Jones et al., 1982). This increases the difficulty of breeding resistant varieties and hinders research on resistance mechanisms of host against *P. brassicae*. In response to these challenges, isolation of a single spore from populations of *P. brassicae* is an effective method. Accurate identification of the pathotype of *P. brassicae* populations is essential for screening effective sources of genetic resistance. Additionally, the successful isolation of single spores is important for pathotype identification efforts (Klewer et al., 2001; Xue et al., 2008).

Methods for single spore isolation of *P. brassicae* are diverse and constantly being improved and optimized. The primary objective is to successfully obtain single spore isolates and enhance the success rate of single spore inoculation. A report in 1977 showed that single spores of *P. brassicae* were cultivated using liquid paraffin droplets as a medium and were successfully inoculated onto Brassica plants, although only 2 out of 250 inoculations were successful (Buczacki, 1977). Voorrips (1996) prepared a suspension of *P. brassicae* spores at a concentration of 500 spores/ml, but the success rate of inoculation was only 1.2%. Xue et al. (2008) prepared a spore suspension containing 5% glycerol to avoid spore aggregation and then inoculated the droplets onto the roots of cabbage seedlings that had germinated for 1–2 weeks. The seedlings were placed in petri dishes lined with filter paper and then incubated at 21°C and darkness for 2d before being transplanted into nutrient pots and incubated at 24°C in light and 18°C in darkness, and the success rate of inoculation was up to 16.95%. These studies were conducted by preparing highly diluted spore suspensions of *P. brassicae* and using droplet inoculation to ultimately obtain single spores. However, there are obstacles that cannot be ignored with the droplet approach to spore suspensions. For example, it is difficult to demonstrate that there is only one microscopic spore in a small droplet, and there may be problems with the absence of a single spore or multiple spores in a droplet. The surface tension of the droplet hinders the identification of stationary spores. In addition, small droplets evaporate easily, whereas large droplets are difficult to identify under high magnification (Askarian et al., 2021; Diederichsen et al., 2016).

Another major method for isolating of single spores of *P. brassicae* was performed using agar as a medium. Tinggal and Webster (1981) developed a technique to inoculate *P. brassicae* with agar in a petri dish, which had a higher success rate relative to the paraffin drop method. Follow-up studies have continually improved this method, resulting in the optimization of the technique for single spore isolation and continued improvement in the success of inoculation (Askarian et al., 2021; Jones et al., 1982). Follow-up studies have continually improved this method, resulting in the optimization of the technique for single spore isolation with success rates of up to 30% (Askarian et al., 2021; Jones et al., 1982). Somé et al. (1996) aspirated a diluted suspension of *P. brassicae* onto an agar block, and the agar block containing one spore was picked out with a needle by microscopic observation and then inoculated into the host root with a success rate of about 9.6%. Despite the high success rate of isolating single spore inoculations of *P. brassicae* using agar-based methods, there are still some limitations to this method. For example, special equipment and micromanipulators are necessary to cut and lift agar gel blocks that contain a single spore (Askarian et al., 2021). In addition, agar tends to solidify and agar blocks are prone to bubbling and can have problems with impurities, which can affect the efficiency and accuracy of single spore isolation.

In addition to these two isolation methods, researchers are continually exploring other techniques to isolate single spores of *P. brassicae*. Diederichsen et al. (2016) inoculated infected root hairs containing a single sporangiosorus of *P. brassicae* onto Brassica seedlings, and successfully obtained single pathotype isolates with a highest infection rate of 17.9%. Recently, a newly developed and efficient systematic protocol for isolation of single spores of *P. brassicae* could increase the rate of infection success up to 47.38%. The procedure is as follows: inoculation of 2-day-old seedlings in cryoboxes, addition of nutrient solution for culture, and microscopic observation of single spores prior to medium incubation (Lv et al., 2021).

Although the *P. brassicae* extracted from single spores is considered genetically homogeneous and it has been shown that single spores are genetically stable (Heo et al., 2009). However, the possibility of introducing heterogeneity in the reproduction process of *P. brassicae* still cannot be ruled out (Askarian et al., 2021). Graf et al. (2004) performed electrophoretic karyotyping of single spore isolates of *P. brassicae* using contour-clamped homogeneous electric field gel electrophoresis and found that they had chromosomally polymorphic. Therefore, the single spore isolation method was unable to obtain consistent and accurate DNA information. In addition, Sharp et al. (2018) sequenced the genomes of more than 200 replica lines of yeast (*Saccharomyces cerevisiae*) and analyzed the number, types, locations, and effects of thousands of mutations. They found that haploids were more prone to single-nucleotide mutations and mitochondrial mutations, while larger structural changes were more common in diploids (Diederichsen et al., 2016).

## 5 Management strategy of *Plasmodiophora brassicae*

As a soil-borne disease, *P. brassicae* is highly insidious and difficult to detected in the early stages of the disease. In addition, it is highly contagious and can spread rapidly with the movement of farm machinery operated in infested field, and can also be transmitted with wind-raised dust and seed translocation (Cao et al., 2009; Gossen et al., 2015; Rennie et al., 2015). The management of clubroot disease has always posed a significant challenge, and various measures have been used to control this disease. However, the implementation of these methods has demonstrated that none are sufficient to completely eradicate the pathogen (Struck et al., 2022).

Due to the variations in pathotypes of *P. brassicae* populations, as well as differences in environmental conditions, farming patterns, and control policies across regions, it is challenging to establish a universally applicable and effective strategies for controlling clubroot. The development of locally adapted integrated strategies for controlling clubroot, tailored to the specific conditions of different regions, is a key focus for future efforts. We summarized various management strategies for *P. brassicae* based on previous studies (Figure 3).

**Figure 2:**
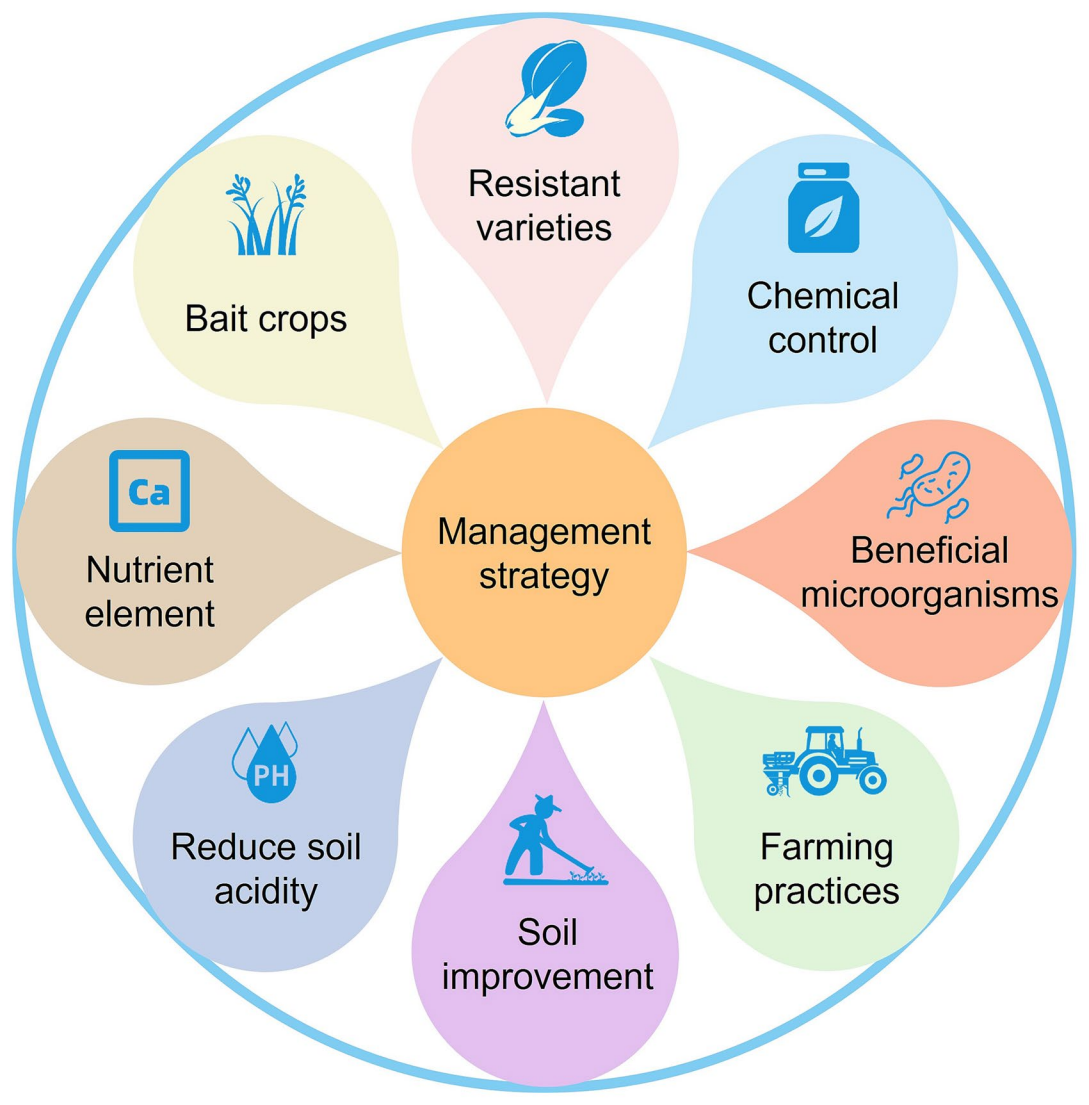
Management strategy of *Plasmodiophora brassicae*.

### 5.1 Planting resistant varieties

Developing and cultivating resistant varieties is the most economical, effective and safe sustainable clubroot management strategy. The development of genetically resistant cultivars can mitigate the harmful effects of *P. brassicae* and reduce crop yield losses (Dolatabadian et al., 2022; Piao et al., 2009). *Brassica* plants consist mainly of three diploid basic species, including *B. rapa* (AA, 2n = 20), *B. nigra* (BB, 2n = 16), and *B. oleracea* (CC, 2n = 18) (Zhu et al., 2022). The genomes of all three of these basic species are likely to contain clubroot resistance genes. Hu et al. (2024) incorporated the clubroot-resistance gene *Rcr1* into clubroot-susceptible elite canola lines using a modified CRISPR/Cas9-based intragenic vector system to develop selection-marker-free canola germplasms with a stable resistant phenotype in <2 years.

The development of technologies such as high-throughput sequencing, genomics, and molecular genetics has greatly promoted the rapid identification and application of clubroot resistance genes (Liégard et al., 2019). It has been reported that resistance in the A genome is usually controlled by a major dominant gene, whereas resistance in the C genome is usually regulated by multiple quantitative trait loci (Hasan et al., 2021). The A genome species contains numerous specific, single, dominant resistance genes and serves as the primary source of resistance genes for *P. brassicae*. Most resistance genes in the species with the C genome consist of a combination of major and minor genes, exhibiting a high degree of continuity (Dolatabadian et al., 2022; Neik et al., 2017). However, the number of resistance genes in the B genome is significantly lower than those in the A and C genomes. It was not until 2019 that the first single dominant gene for clubroot resistance, *Rcr6*, was identified. This gene is located on the B3_Canada chromosome and is homologous to chromosome A08 in the A genome (Chang et al., 2019). The identified clubroot resistant genes and their mapped chromosomes are summarized in Table 3.

**Table 3:** List of identified clubroot resistant genes to *Plasmodiophora brassicae* and their mapped chromosomes.

Clubroot resistant genes	Accession numbers	Chromosome	Cultivar or line	References
*Cra*	AB751516	A3	ECD02	Matsumoto et al. (1998)
*CRb*	LC155800.1	A3	Shinki	Piao et al. (2004)
*Crr3*	a	A3	Milan White	Hirai et al. (2004)
*Rcr1*	–	A3	Pak choy	Chu et al. (2014)
*Rcr2*	–	A3	Chinese cabbage cv. “Jazz”	Huang et al. (2017)
*Rcr4*	–	A3	Pluto	Yu et al. (2017)
*Rcr5*	–	A3	Purple Top White Globe	Huang et al. (2019)
*Rcr8*	–	A3	Pluto	Yu et al. (2017)
*Rcr9*	–	A3	Pluto	Yu et al. (2017)
*CRk*	–	A3	Debra	Sakamoto et al. (2008)
*CRd*	–	A3	Chinese cabbage	Pang et al. (2018)
*PbBa3.1*	–	A3	ECD04	Chen et al. (2013)
*PbBa3.2*	–	A3	ECD04	Chen et al. (2013)
*PbBa3.3*	–	A3	ECD04	Chen et al. (2013)
*BraA.CR.a*	–	A3	ECD01, ECD02, ECD04	Hirani et al. (2018)
*BraA.CR.c*	–	A3	ECD03	Hirani et al. (2018)
*BraA 3P5X.Cra/b^Kato^ 1.1*	–	A3	ECD02	Fredua-Agyeman et al. (2020)
*BraA 3P5X.Cra/b^Kato^ 1.2*	–	A3	ECD02	Fredua-Agyeman et al. (2020)
*CRA3.7*	–	A3	Chinese cabbage line CR510	Pang et al. (2022)
*CRq*	LC155799.1	A3	Chinese cabbage DH line Y635-10	Wei et al. (2022)
*Rcr10^ECD01^*	*BnaA03g25330D*	A3	*Brassica rapa* turnip cv. ECD01	Yu et al. (2022)
*qCRa3–1*	–	A3	*Brassica juncea* line CT19	Li K. Q. et al. (2024)
*Crr1*	AB605024.1	A8	Siloga	Suwabe et al. (2003)
*Rcr3*	–	A8	ECD04	Karim et al. (2020)
*Rcr9^wa^*	–	A8	ECD04	Karim et al. (2020)
*CRs*	–	A8	SCNU-T2016	Laila et al. (2019)
*PbBa8.1*	–	A8	ECD04	Chen et al. (2013)
*BraA.CR.b*	–	A8	ECD01, ECD02, ECD03, ECD04	Hirani et al. (2018)
*PbBrA08^Banglim^*	–	A8	09CR500	Choi et al. (2020)
*Cr4Ba8.1*	–	A8	Bap246	Zhang H. et al. (2024)
*CRA8.1*	–	A8	Cabbage material DingWen	Wang et al. (2022)
*Rcr9^ECD02^*	–	A8	*Brassica rapa* turnip ECD02	Rahaman et al. (2022)
*Rcr9^ECD01^*	*BnaA08g10100D, BnaA08g11840D*	A8	*Brassica rapa* turnip cv. ECD01	Yu et al. (2022)
*BnaA8P3D.CRX1.1*	106,396,583	A8	*Brassica napus* ssp. *napobrassica* FGRA106	Yu et al. (2024a)
*BnaA8P3D.RCr91.2*	106,418,916	A8	*Brassica napus* ssp. *napobrassica* FGRA106	Yu et al. (2024a)
*BnaA8P3H.Crr11.3*	106,361,033, 106,361,045, 106,361,049, 106,361,048	A8	*Brassica napus* ssp. *napobrassica* FGRA106	Yu et al. (2024a)
*BnaA8P3H.qBrCR381.4*	106,361,308, 106,361,304, 106,361,282, 106,405,582, 106,361,258	A8	*Brassica napus* ssp. *napobrassica* FGRA106	Yu et al. (2024a)
*Crr2*	–	A1	Siloga	Suwabe et al. (2003)
*PbBa1.1*	–	A1	ECD04	Chen et al. (2013)
*Cr4Ba1.1*	–	A1	Bap246	Zhang H. et al. (2024)
*CRc*	–	A2	Debra	Sakamoto et al. (2008)
*BnaA5P3A.CRX1.1*	106,362,025	A5	*Brassica napus* ssp. *napobrassica* FGRA106	Yu et al. (2024a)
*Crr4*	103,866,882	A6	Siloga	Suwabe et al. (2006)
*Rcr6*	–	B3	*Brassica nigra* line PI 219576	Chang et al. (2019)
*BnaC1P3H.CRX1.2*	106,349,084, 106,349,049	C1	*Brassica napus* ssp. *napobrassica* FGRA106	Yu et al. (2024a)
*Crs1*	–	C	*Raphanus sativus* L.	Kamei et al. (2010)
*Rcr7*	–	C7	*Brassica oleracea* cultivars “Tekila”	Dakouri et al. (2018)
*CR2a*	–	C	*Brassica oleracea* line no. 86–16-5	Landry et al. (1992)
*CR2b*	–	C	*Brassica oleracea* line no. 86–16-5	Landry et al. (1992)
*pb-3*	–	C	*Brassica oleracea* DH line Geman landrace Bindsachsener	Voorrips et al. (1997)
*pb-4*	–	C	*Brassica oleracea* DH line Geman landrace Bindsachsener	Voorrips et al. (1997)
*PbBo1*	–	C	*Brassica oleracea* (Landrace kale line “C10”)	Rocherieux et al. (2004)
*Rcr_C03-1*	–	C3	*Brassica oleracea* cultivar ECD11	Karim and Yu (2023)
*qCRc7-2*	–	C7	*Brassica oleracea* line “GZ87”	Ce et al. (2021)
*qCRc7-3*	–	C7	*Brassica oleracea* line “GZ87”	Ce et al. (2021)
*qCRc7-4*	–	C7	*Brassica oleracea* line “GZ87”	Ce et al. (2021)
*BnaC7P3A.CRX1.1*	106,418,155/106418157	C7	*Brassica napus* ssp. *napobrassica* FGRA106	Yu et al. (2024a)
*Rcr_C08-1*	–	C8	*Brassica oleracea* cultivar ECD11	Karim and Yu (2023)
*BolC.Pb9.1*	–	C9	Wild *Brassica oleracea* B2013	Zhang X. L. et al. (2024b)
*Pb-At5.2*	–	5	*Arabidopsis accessions* line *Bur-0*	Gravot et al. (2024)

^aDashes indicate unknown.^

Most clubroot resistance genes have been identified in *Brassica* plants, although other cruciferous species also possess the potential genetic resources for resistance. Wang et al. (2023) reported a broad-spectrum clubroot resistance gene, *WeiTsing* (*WTS*), isolated from Arabidopsis, which is induced to up-regulate its expression in the pericycle upon *P. brassicae* infection to prevent the pathogen colonization in the stele. In addition, *WTS* encodes a small protein localized in the endoplasmic reticulum that oligomerizes to form a pentameric cation-selective ion channel permeable to Ca^2+^. The infestation of *P. brassicae* can activate this calcium-permeable channel and thereby triggering plant immunity.

At present, there are numerous detailing the mechanisms by which resistant varieties against *P. brassicae*. Most studies have revealed that *P. brassicae* can be recognized and triggers a complex series of immune responses when infesting resistant varieties (Mei et al., 2019; Ning et al., 2019; Ochoa et al., 2023). With the development of bioinformatics, multiple omics technologies have made certain progress in elucidating resistance mechanisms. This has not only allowed us to gain a deeper understanding of the dynamic changes that transpired during host-pathogen interactions, but also provided us with a wider perspective to explore the potential mechanisms of action (Ludwig-Müller, 2022; Shaw et al., 2022). A recent study revealed the resistance mechanism of the cabbage variety “Shangpin” with broad-spectrum immunity to *P. brassicae*. It may enhance disease resistance by recruiting beneficial microorganisms, such as *Flavobacterium* and *Sphingomonas*, to maintain the stability of the root microbial community structure while inhibiting the growth and reproduction of *P. brassicae* (Fang et al., 2024).

However, most sources of resistance are specific to the *P. brassicae* prototype and the main resistant varieties circulating on the market are single-gene, which makes resistance of these varieties not durable. In particular, the selection pressure exerted by *P. brassicae* populations on resistant varieties produces changes in virulence that may lead to loss of resistance (Botero-Ramírez et al., 2021). Faced with the pressure of resistance of from resistant varieties being overcome by *P. brassicae*, the primary coping strategies can be worked from the following ways. On the one hand, new resistance genes should be continuously explored and new resistant varieties should be developed. On the other hand, the polymerization of two or more resistance genes to develop superior resistant varieties. Before these resistance genes can be utilized in breeding efforts, their characteristics and relationships must be evaluated in detail. Li et al. (2022) used a marker-assisted selection method to polymerize two resistance genes, *CRa* and *CRd*, into Chinese cabbage to develop homozygous pyramided lines that showed strong resistance to six different pathotypes of *P. brassicae*.

### 5.2 Chemical control

Chemical control is a crucial and effective method for managing clubroot disease in the field and is the primary choice for growers aiming to combat this disease. There are two main types of chemical agents used to control *P. brassicae*: namely fungicides and fumigants. To date, efficacy assessments have been conducted for a wide range of chemicals, including fungicides and fumigants. Detailed information on the effectiveness of different chemicals against *P. brassicae* is shown in Table 4.

**Table 4:** Summary of chemical agents for controlling *Plasmodiophora brassicae.*

Type	Chemical agent	Host plant	Control effect	References
Fungicide	Fluazinam	Chinese cabbage	Control efficiency of field trial was 98.72%	Liu et al. (2019)
	Cyazofamid	*Bassica rapa* subsp. *chinensis* var. *communis* and *Bassica rapa* subsp. chinensis var. *utilis*	Inhibited root hair infection and club formation	Gossen et al. (2012)
	Flusulfamide	*Brassica rapa*	Inhibiting germination	Sasaki et al. (2024)
	Amisulbrom	*Brassica napus*	Inhibited resting spore germination by up to 79% and reduced viable spores by 31%	Yu et al. (2024b)
	Benomyl	*Bassica campestris* ssp. *pekinensis* cv.	Inhibited root hair infection, in reducing club development and inhibited secondary ascospore colonization	Naiki and Dixon (1987)
	Pentachloronitrobenzene	*Bassica campestris* ssp. *pekinensis* cv.	Inhibited spore germination	Naiki and Dixon (1987)
Fumigant	Ethanedinitrile	*Bassica rapa* L. ssp. *Pekinensis*	Inhibited clubroot disease by 81.39%	Patar et al. (2023)
	Dazomet	Canola cultivar 45H26	Reduced the severity of clubroot	Hwang et al. (2018a)
	Metham sodium	*Brassica napus*	Reduced root hair infection, gall weight and clubroot severity under greenhouse conditions	Hwang et al. (2018b)
	Chloropicrin	Cauliflowers	Reduced the population density of *Plasmodiophora brassicae* and disease rating	Porter et al. (1991)

Fungicides are an attractive option for clubroot management. A range of fungicides have been identified as effective against *P. brassicae*, but only a few have been registered. It has been reported that benomyl and pentachloronitrobenzene can reduce root-hair infection and clubroot development (Naiki and Dixon, 1987). Flusulfamide has been registered as a fungicide against clubroot in several countries. It adsorbs to the cell walls of resting spores to inhibit their germination. The mechanism of action has been reported to involve the inhibition of germination by inducing the overexpression of the immunophilin gene *PbCyp3*. This overexpression leads to an unusual accumulation of the PbCYP3 protein, which causes aberrant folding of the proteins involved in primary zoospores (Sasaki et al., 2024; Tanaka et al., 1999). Fluazinam is a broad-spectrum fungicide developed by Ishihara Sangyo Kaisha Ltd. of Japan, which belongs to chemical group of the 2,6-dinitroanilines. Liu et al. (2019) found that fluazinam reduced the disease index of clubroot to 0.95% and provided 98.72% control efficiency of *P. brassicae*. Liao et al. (2022) reported a control efficacy of 59.81% against clubroot disease using fluazinam, along with a 21.29% reduction in the abundance of *P. brassicae* in the soil after 3 weeks of treatment. Cyazofamid effectively inhibits root hair infection and club formation by directly inhibiting the germination of resting spores (Mitani et al., 2003). In the presence of high inoculum pressure, the drench application of cyazofamid was shown to substantially reduce the incidence and severity of clubroot in Shanghai pak choy and Chinese flowering cabbage (Gossen et al., 2012). Amisulbrom is a quinone inside inhibitor. *In vitro* experiments have shown that it inhibits resting spore germination by up to 79% and reduces spore viability by 31%. In addition, application of amisulbrom under both greenhouse and field conditions significantly reduced the clubroot severity (Yu et al., 2024b).

Soil fumigation prior to crop planting is an effective and reliable method for controlling soil-borne diseases and alleviating the obstacles to continuous cropping. Methyl bromide, an earlier fumigant used for clubroot control, has been restricted due to its deleterious effects on ozone concentrations (Ruzo, 2006; White and Buczacki, 1977). Previous studies have reported that chloropicrin is effective in reducing clubroot, however, it has been banned in several countries due to potential risks to non-target organisms and human health (Pesonen and Vähäkangas, 2020; Porter et al., 1991). Dazomet, a solid fumigant, reduces the severity of clubroot under both greenhouse and field conditions, but excessive concentrations (e.g., 0.4–0.8 t a.i. ha^−1^) can lead to a reduction in seedling emergence and yield (Hwang et al., 2018a). Metham sodium is a broad-spectrum dithiocarbamate fumigant that rapidly decomposes to produce biologically active volatile methyl isothiocyanate when applied to soil. The application of 0.4 to 1.6 mL of metham sodium per liter of soil under greenhouse conditions has been shown to effectively reduce root hair infections, gall weight, and clubroot severity, while also improving the health of canola crops (Hwang et al., 2018b). It is important to note that metham sodium has limited mobility in the soil and requires adequate fumigation time, as well as appropriate soil moisture and temperature. Ethanedinitrile, a cyano-fumigant, was shown in a recent study to inhibit clubroot disease by 81.39%, with complete control of the disease when treated for 12 h at rates of 42 and 50 g/m^3^ or as well as at a rate of 35 g/m^3^ for 48 h (Patar et al., 2023).

Despite the fact that chemicals have certain advantages in terms of disease control effectiveness, there are also limitations that cannot be ignored. The most notable limitations are the adverse effects of exposure to the ecosystem and the potential risks to human health (Zhang J. H. et al., 2024). Moreover, chemicals do not offer advantages in terms of production costs, and their lower cost-effectiveness is a major limiting factor. Chemicals are suitable for the control of clubroot disease in high-yield facility vegetable crops. However, for lower-value field crops such as oilseed rape, neither chemical fungicides nor chemical fumigants are typically the preferred options for growers (Zuzak et al., 2023). In addition, the control effect of chemicals is susceptible to receive the influence of many factors such as spore number, physiological pathotypes, application method and soil temperature, moisture, pH, and weather conditions (Hwang et al., 2014a; Zuzak et al., 2023).

### 5.3 Beneficial microorganisms

The use of microbial resources for clubroot control is an economical, ecologically friendly, and sustainable management strategy that is considered an alternative to chemical control. In recent years, there has been an increasing interest in utilizing beneficial microorganisms as biological control agents to control plant diseases. The great potential of antagonistic microorganisms in reducing clubroot severity and inhibiting *P. brassicae* infestation has been demonstrated in numerous studies. In addition, the ability of microorganisms to colonize the soil or within the plant may provide durable protection against *P. brassicae* damage (Dombrowski et al., 2017). Microorganisms control pathogens through various mechanisms, mainly including antibiosis, parasitism, production of active substances, induction of plant resistance and or competition for resources (Xu et al., 2011). Detailed information on the efficacy of different microbial strains against *P. brassicae* is shown in Table 5.

**Table 5:** Summary of microbial strains for controlling *Plasmodiophora brassicae.*

Microbial species	Strains	Biocontrol activity	Application method	References
Bacillus	*Bacillus subtilis* QST713	Reduced the clubroot severity in canola by 62–83%	Soil drench	Lahlali et al. (2013)
	*Bacillus subtilis* XF-1	Reduced disease incidence rate and severity of clubroot by 40 and 69%	Soaked seed with culture	He et al. (2019)
	*Bacillus cereus* MZ-12	Repressed root hair infection	Soaked seedlings	Arif et al. (2021)
	*Lysobacter antibioticus* YFY 02, 13–1, and HY	Reduced clubroot severity on Chinese cabbage by 37.7–74.6% and 62.4–85.1% under greenhouse and field conditions	Soil drench	Zhou et al. (2014)
	*Streptomyces platensis* 3–10	Inhibit the germination of resting spores by 75 and 80%	Added crude extract solution of cultural filtrates to resting spore suspension	Shakeel et al. (2016)
	*Streptomyces melanosporofaciens* X216	Control effects on clubroot in greenhouse and field test were 62.14 and 43.16%	Soil drench	Ding et al. (2023)
Fungi	*Acremonium alternatum*	Reduced the disease index by 50%	*Acremonium alternatum* spores or spore extract were added to the tubes containing resting spores with and without roots	Jäschke et al. (2010)
	*Heteroconium chaetospira* BC2HB1	Induced plant resistance	Granular formulation of BC2HB1 was mixed with planting mix	Lahlali et al. (2014)
	*Piriformospora indica*	Reduced the disease index by 61.60%	Experimental plants were inoculated with *Piriformospora indica*	Khalid et al. (2020)
	*Trichoderma guizhouense* Hz36 and Hz37	The biocontrol efficiency on *Brassica napus* clubroot disease was 57.30%, and the control effect on *Arabidopsis thaliana* clubroot was 68.01%	Co-inoculated the plant with *Plasmodiophora brassicae* and Hz36 or Hk37	Zhao et al. (2022)
	*Trichoderma viride* TR-7	Reduced spore germination	TR-7 spores or a spore extract were added to resting spores including roots and without roots in tubes	Arif et al. (2023)

Currently, a large number of investigations have revealed that bacterial biocontrol strains have a controlling effect on *P. brassicae*. Serenade, a biofungicide consisting of *Bacillus subtilis* QST713, when applied as a soil drench, has been reported to reduce the clubroot severity in canola by 62–83% (Lahlali et al., 2013). *B. subtilis* XF-1 has strong inhibitory effects on both the survival and germination of dormant spores of *P. brassicae*. Seed soaking with a culture of XF-1 reduced disease incidence rate and severity of clubroot by 40 and 69%, respectively (He et al., 2019). Arif et al. (2021) isolated a strain of the endophytic bacterium *B. cereus* MZ-12 from rhizosphere soil of pak choi that was able to colonize cabbage roots to inhibit *P. brassicae* infestation, and also inhibited infestation through direct inhibition of zoospores. Zhou et al. (2014) screened three strains of *Lysobacter antibioticus*, YFY 02, 13–1, and HY, from vegetable rhizosphere soils. The cell-free culture filtrates of these strains reduced clubroot severity on Chinese cabbage by 37.7–74.6% under greenhouse conditions and by 62.4–85.1% under field conditions. There are also a number of streptomycetes that have been tapped for their potential to control clubroot. The culture conditions of *Streptomyces platensis* 3–10 were optimized to inhibit the germination of resting spores by 75 and 80% for its culture filtrates and crude extracts, respectively (Shakeel et al., 2016). Ding et al. (2023) isolated a strain of *S. melanosporofaciens* X216 from fields of oilseed rape, which showed a lethality of 56.59% against resting spores, and was effective in controlling clubroot disease of oilseed rape by 62.14% in greenhouse pots and 43.16% and under field conditions, respectively.

Fungi are another group of microorganisms that have been extensively investigated for *P. brassica* control. Jäschke et al. (2010) found that inoculation of *A. thaliana* with both *P. brassicae* and the endophytic fungus *Acremonium alternatum* at the same time induced a 50% reduction in the disease index compared to inoculation with *P. brassicae* only. The endophytic fungus *Heteroconium chaetospira* has been reported to penetrate canola roots and colonize the cortical tissues, promoting the up-regulated expression of genes involved in the biosynthesis of jasmonic acid, ethylene, and auxin, thereby inducing resistance to *P. brassicae* in canola (Lahlali et al., 2014). *Piriformospora indica* was able to colonize *B. campestris* roots, and co-inoculation with *P. indica* and *P. brassicae* resulted in a 61.6% reduction in the disease index of the plant compared to inoculated with *P. brassicae* only (Khalid et al., 2020). Zhao et al. (2022) screened two *Trichoderma* strains, *T. guizhouense* (Hz36) and *T. koningiopsis* (Hz37), which significantly inhibited the germination of resting spores. The biocontrol efficiency of 44.29 and 57.30% against clubroot in oilseed rape, respectively, and for the biocontrol efficiency of 52.18 and 68.01% in *A. thaliana*, respectively. A strain of *T. viride* TR-7 was isolated from tomato rhizosphere soil, which could reduce the germination rate of resting spores to 25.3%. Co-inoculation with TR-7 and *P. brassicae* resulted in a 56.7% reduction in gall growth compared to pak choi treated with *P. brassicae* alone (Arif et al., 2023).

However, a major challenge in the use of microorganisms to control soil-borne diseases is their limited stability. The biological activity of antagonistic microorganisms may be diminished or rendered ineffective by various of environmental factors, such as temperature, humidity, pH, and light (Nicholson, 2002). To solve this dilemma, plant pathologists are constantly searching and experimenting with methods to protect and enhance the biological activity of microorganisms. Encapsulation or embedding of microorganisms using biocompatible materials is an effective strategy. Pelletization of cabbage seeds with microencapsulated *Paenibacillus polymyxa* ZF129 has been reported to enhance stability and improve bioactivity against clubroot disease under greenhouse conditions, achieving a control efficacy of 71.23% (Abdukerim et al., 2023). Kang et al. (2024) prepared macrobeads embedded with *P. polymyxa* ZF129 (chitosan: carrageenan, 1:1) using ionotropic gelation. This method enhanced stability compared to free bacteria and showed higher control efficacy in controlling clubroot disease than the ZF129 culture both in greenhouse and field conditions. Another promising solution is to combine different microbial strains into a microbial consortium. Microbial consortia consisting of two or more beneficial microbial strains are better adapted to the pressures of the rhizosphere environment and are more effective in suppressing soil-borne pathogens when the compatibility and modes of action of the microbial strains are carefully evaluated (Niu et al., 2020). A field trial revealed that the microbial consortia of *B. cereus* BT-23, *L. antibioticus* 13–6, and *L. capsici* ZST1-2 reduced the incidence of clubroot in Chinese cabbage by decreasing soil acidity and reshaping the diversity and structure of the rhizobial community, achieving a biocontrol effect of 65.78% (Zhang et al., 2022).

### 5.4 Farming practices

Agricultural producers recognized early on that some farming practices, such as the timely removal of diseased plants, improved drainage, and deep plowing and tilling, could reduce the infestation rate of *P. brassicae* or the severity of clubroot disease (Botero et al., 2019). It should be noted that infected seeds are an important pathway leading to the long-distance dissemination of *P. brassicae*, making seed cleaning particularly important (Greer et al., 2023). However, they often ignore the fact that the resting spores can easily spread in the field with the soil adhering to agricultural machinery and their own boots (Gossen et al., 2013a).

Crop rotation is a time-honored farming practice that helps maintain soil structure and organic matter, reduces soil erosion, and also reduces plant diseases caused by soil-borne pathogens (Janvier et al., 2007). Selection of appropriate crop rotations must begin with an understanding of the host range of the pathogen. To reduce the risk of plant diseases, developing effective rotation patterns necessitates the selection of non-host crops, the design of rational rotation sequences, and the establishment of appropriate intervals (Krupinsky et al., 2002). Compared to Chinese cabbage monoculture, rotating of cabbage with potato onion reduced the disease incidence and disease index of clubroot by 34.3 and 37.5%, respectively (Chen et al., 2018). Yang et al. (2020b) selected leguminous crops (soybean, clover), gramineous crops (rice, maize) and cruciferous crops (oilseed rape, Chinese cabbage) as preceding crops to rotate with oilseed rape. They found that the soybean-oilseed rape rotation pattern was the most effective for controlling clubroot, resulting in the lowest density of resting spores in the soil (< 2.0 × 10^6^ spores per gram of soil). This rotation led to a 50% reduction in the incidence rate and a 40% decrease in the disease index.

Weather conditions, particularly temperature and rainfall, vary considerably depending on the seeding dates. It is well known that soil temperature and moisture play a crucial role in the development of clubroot. Therefore, different seeding dates are bound to have different effects on rhizoctonia. Gossen et al. (2012) showed that seeding date had a significant effect on clubroot incidence and severity on both Shanghai pak choy and Chinese flowering cabbage. Therefore, the risk of clubroot can be minimized by choosing an appropriate seeding date to avoid the optimum temperature conditions required by *P. brassicae*. A field experiment conducted in Ontario, Canada, showed that temperature and rainfall influence the germination and development of *P. brassicae.* Additionally, seeding brassica crops as early as possible in the region can reduce the severity of clubroot (Gossen et al., 2017).

The use of solarization combined with mulching is also a management method to control clubroot in agricultural practices. Hong et al. (2023) evaluated the effect of plastic mulching on clubroot under greenhouse and field conditions. The results showed that mulching increased the soil temperature in the 0–20 cm layer and reduced the *P. brassicae* population in the soil, which effectively reduced the incidence and severity of clubroot.

Cutting off transmission route of pathogens is one of the basic strategies for controlling clubroot. Although the possibility of *P. brassicae* entering clean fields has been reduced by cultural controls, such as maintaining farm and nursery sanitation and seed disinfection. However, the reality is that the rate of spread speed and extent of infestation of *P. brassicae* are still increasing, and it is difficult to achieve complete interruption of the spread of *P. brassicae*. In addition, conventional farming practices have limitations in controlling clubroot disease, especially when confronted with excessive inoculum pressure, and these measures are often unsatisfactory (Peng et al., 2014).

### 5.5 Soil improvement

Appropriate amelioration of infested soils not only improves soil properties but also benefits the control of soil-borne diseases, making it a promising strategy for improving agricultural sustainability. The benefits of using soil amendments to control soilborne diseases are gradually apparent, usually slower in onset but longer in duration, and the effects can be cumulative (Bailey and Lazarovits, 2003). In addition, soil amendments can directly or indirectly influence the balance between beneficial and detrimental microbial populations in the soil. They achieve this by enhancing the biomass, activity, diversity, and structure of soil microorganisms, which in turn improves the general suppression of pathogens in the soil (Chen et al., 2020). Zhang et al. (2019) found that soil amendments, including quicklime and organic fertilizers, altered soil pH and available nutrients. These amendments also enhanced the relative abundance of bacteria with biocontrol potential, such as *Xanthomonadales*, *Pseudomonas*, and *Bacillus*, as well as increased the enzyme activities of urease and polyphenol oxidase in the topsoil of Chinese cabbage fields.

There is a wide range of soil amendments commonly used in agricultural production, mainly including various organic amendments (e.g., chemical fertilizers, animal manure, solid waste and composts) and crop residues. Calcium cyanamide (CaCN_2_) is a time-honored and slow-release nitrogen fertilizer that not only provides a source of nitrogen and carbon for crops but also produces degradation products that increase soil pH (Dixon, 2017). Therefore, calcium cyanamide is widely used as a soil amendment to manage clubroot disease. A study conducted in Australia assessed the efficacy of calcium cyanamide against clubroot disease under field and greenhouse conditions. The results indicated that broadcasting 1,000 kg/ha of small particles (98% w/w < 300 μm) of calcium cyanamide effectively controlled clubroot disease (Donald et al., 2004). Niwa et al. (2007) conducted a seven-year continuous field trial and highly reproducible bioassay experiments, demonstrating that the application of farmyard manure (compost mixed with cattle feces and rice straw) and food factory sludge compost (compost mixed with dehydrated activated sludge and corn gluten feed discharged from a cornstarch factory) effectively suppressed clubroot. The main mechanism of action was identified as the calcium enrichment of organic fertilizers, which led to an increase in soil pH.

The utilization of crop residues is an important measure of residue management in sustainable agricultural development and serves as one of the main sources of organic matter in agricultural soils. Straw is a natural soil amendment material commonly used in agricultural production and is a severely underestimated source of organic carbon. It not only plays an important role in regulating the soil environment but also contributes positively to the control soil-borne diseases (Hu et al., 2023). A recent study showed that applying rice straw enhanced the level of available nutrients, pH, and electrical conductivity in the soil of Chinese cabbage field. However, it also decreased the alpha diversity of the bacterial community and altered its community composition, which helped to suppress the incidence of clubroot disease (Han et al., 2021). Similarly, a two-year field trial showed that maize, rice, and wheat straw all promoted the growth of Chinese cabbage plants, improved the rhizosphere microbiome, and reduced the abundance of *P. brassicae* (Di et al., 2023).

The purpose of soil amendment is to maintain soil health, with the suppression of pathogens serving as a crucial indicator of soil health. Soil amendments have a significant impact on soil health, including plant health, by altering soil physicochemical properties and influencing soil microbial communities. However, the inhibitory activity of soil is a complex process influenced by many biotic and abiotic factors, and the mechanism underlying the inhibitory properties are not yet fully understood. In addition, the application of soil amendments has been shown to have varying effects on soil-borne diseases. Although reports on the inhibitory properties of soil amendments against pathogens are generally encouraging, issues such as reduced or failing inhibitory activity, as well as inconsistency and unpredictability, continue to pose significant challenges to the application of soil amendments (De Corato, 2023). Previous studies have demonstrated the variable control efficacy of soil amendment applications in different regions and under different levels of disease pressure, highlighting the limitations of relying solely on this control measure (McGrann et al., 2016).

### 5.6 Reduce soil acidity

*P. brassicae* usually grows in acidic soils environments, which promotes the germination of resting spores when the soil pH ranges from 5 to 7 (Rashid et al., 2013). Previous studies have revealed that both pH and calcium content significantly impact the longevity and germination of resting spores. Additionally, these factors strongly influence on the primary infestation stage of *P. brassicae* and colonization of the root hairs (Dixon, 2014). Bhering et al. (2017) found a direct relationship between soil acidity on reduction of the healthy root volumes of Brazilian cauliflower and increase of the root volumes with clubroot. For this reason, reducing soil acidity is beneficial in inhibiting the development of *P. brassicae*. Controlling clubroot by lowering soil acidity is one of the oldest and most widely used management strategies. The application of lime-based substances has been the most commonly used measure to achieve this (Dobson et al., 1983).

Previously, there have been numerous reports on the successful application of lime-based products to control the development of clubroot. For example, it has been reported that applying hydrated lime or calcitic limestone dust to raise the pH value of field soil above 7.0 prior to planting cauliflower can effectively reduce the disease indices (Tremblay et al., 2005). Hennig et al. (2022) found that applying hydrated lime to raise soil pH from an initial 5.2–5.5 to 7.2 reduced the severity of clubroot disease in clubroot-susceptible canola cultivar by 34–36%. Additionally, it decreased the density of resting spores by 48–80% and increased yield by 70–98%. Fox et al. (2022) evaluated the control effect of lime products against *P. brassicae* under both greenhouse and field conditions. In greenhouse experiments, the disease index of clubroot in susceptible and resistant oilseed canola cultivars was 0% following the application of hydrated lime [Ca(OH)₂] at doses of 4.7, 8.1, 11.4, and 14.8 tonnes/ha. In contrast, limestone (CaCO₃) only reduced the disease index at inoculum concentrations of <1 × 10^4^ spores g^−1^. In field trials, hydrated lime reduced the clubroot severity. However, control was affected by rainfall and sowing time. Previous studies have indicated that lime can be effective in mitigating clubroot; however, the degree of control afforded may be influenced by the type of lime used, the timing of application, and many environmental and soil factors (Murakami et al., 2002).

However, merely increasing soil pH is not sufficient to control clubroot disease and infestation. This is due to the influence of various of environmental factors, such as nutrient availability, moisture levels, and soil temperature, which all affect the extent of *P. brassicae* infestation and proliferation (McDonald and Westerveld, 2008). Gossen et al. (2013b) evaluated the effects of a wide range of temperature and pH conditions on *P. brassicae* infestation in canola under controlled conditions. They found that applying lime to raise soil pH prevented clubroot disease to some extent; however, moderate to severe clubroot still occurred even at a pH of 8.0, provided that the temperatures were suitable (20–25°C) and soil moisture was adequate.

### 5.7 Nutrient element

Nutrients are necessary for the normal growth and development of plants. They mainly include the massive elements of nitrogen, phosphorus, and potassium; the medium elements of calcium, magnesium; and sulfur; the trace elements of iron, molybdenum, zinc, copper, boron, and manganese. In addition to providing nutritive value to the host, nutrients in the soil environment such as calcium, boron, magnesium, and silicon play an important role in the relationship between the host and clubroot (Dixon, 2009b).

There is a broad consensus on the various roles that calcium plays in inhibiting *P. brassicae*. Earlier studies have demonstrated that calcium inhibits sporangial dehiscence at high inoculum levels of *P. brassicae* spores and also inhibits sporangial development at low inoculum levels (Webster and Dixon, 1991a). A study by Niwa et al. (2008) provided direct evidence that high levels of calcium under neutral conditions can inhibit the resting spore germination of *P. brassicae.* In addition, calcium is essential for the structural strength of cell walls, the normal structure of membranes, and the transport and retention of ions. The stability of cell walls and cell membranes is likely to inhibit the invasion of *P. brassicae* (Donald and Porter, 2009).

Boron is an essential micronutrient for plant growth, and *Brassica* crops, especially oilseed rape, have a high demand for this element. However, excessive application of Boron can result in toxicity symptoms (Brown and Shelp, 1997; Kaur et al., 2006). Numerous studies have revealed that boron plays a significant role in crop inhibition of clubroot (Ruaro et al., 2009; Webster and Dixon, 1991b). Deora et al. (2011) found that under controlled conditions, increased boron application reduced root hair infections as well as primary and secondary infections. However, when applied at rates higher than 2 kg/ha, canola seedlings exhibited symptoms of toxicity. In addition, boron reduced both the incidence and severity of clubroot in the field, with an application rate of 4 kg/ha proving to be the most effective without causing any phytotoxicity symptoms.

Silicon plays a positive role in plant-pathogen interactions by mediating plant resistance to pathogens. It does this by serving as a physical barrier, activating the activity of defense-related enzymes, and regulating the expression of genes involved in the defense response (Wang et al., 2017). A recent study showed that silicon s reduced the disease index of clubroot in spring canola and improved shoot height and root length in spring rape under greenhouse conditions (Sarkar et al., 2023).

In addition to the above several nutrient elements, magnesium ion concentration also has a certain effect on inhibiting clubroot. Myers and Campbell (1985) showed that the infestation of broccoli by *P. brassicae* and the development of clubbing were inhibited when calcium or magnesium concentrations were increased from 0.5 mM to 2.5 mM (Myers and Campbell, 1985).

The interrelationships between nutrient elements in the soil system are very complex. Together with the host and *P. brassicae*, they form a complex nutrient delivery system that plays a crucial role in maintaining material balance and ecological stability within the soil microenvironmental. Although many nutrient elements have been reported to inhibit *P. brassicae*, the mechanisms of action for each element against *P. brassicae* and their interactions with one another remain unclear. This is a huge challenge for future research on nutrient control of clubroot.

### 5.8 Bait crops

The surface of resting spores of *P. brassicae* is covered with a thick shell layer that helps resist most environmental stresses. However, resting spores germinate into primary zoospores, which are short-lived and sensitive to environmental conditions. They can perish quickly if they fail to successfully infect a host. Germination of resting spores is necessary for root infestation by *P. brassicae*. It is generally believed that the root exudates of plants are key factors in inducing the germination of resting spores (Rashid et al., 2013). However, it is worth noting that these root exudates do not exhibit host specificity in stimulating the germination of resting spores; root exudates from non-host plants can also induce spore germination. Friberg et al. (2005) investigated the stimulation of resting spores by the root exudates of the host plant *Brassica rapa* and four non-host plants, finding that the root exudates of *Lolium perenne* exhibited the strongest stimulatory capacity. Some plants can induce germination of resting spores without infection or with only root hair infectionand are considered bait crops (Ren et al., 2016). Typically, bait crops are primarily non-host crops of *P. brassicae*. However, host plants can also be used as bait crops in cases where they are killed prior to resting spore infestation or the formation of clubs (Hwang et al., 2014b).

In the absence of a host, stimulation the germination of resting spore helps accelerate the reduction of spore numbers, as pathogens cannot complete their life cycle without living hosts. Therefore, planting bait crops to stimulate the germination of resting spores without causing infestation is one potential strategy for managing clubroot in large-scale fields. Murakami et al. (2000) found that potted *Raphanus sativus* can reduce the number of resting spores by 71%, and can reduce the number of resting spores in the field by 94%. A recent study demonstrated that, compared to the bare soil control, *Bromus inermis*, *B. riparius*, and *L. perenne* were able to reduce spore concentration by more than 50% under pot conditions. Additionally, *Triticum aestivum*, *Hordeum vulgare*, and *Pisum sativum* decreased spore concentrations by 61, 43, and 39%, respectively (Drury et al., 2022).

However, the actual efficacy and potential of growing bait crops for clubroot control remain inconclusive. Studies have indicated that the impact of bait crops on clubroot severity is minimal and inconsistent (Friberg et al., 2006). Ahmed et al. (2011) evaluated the impact of bait crops, including non-cruciferous host crops (*Trifolium pratense*, *L. perenne*, *Dactylis glomerata*, *Agrotis palustris*) and on-host crops (*H. vulgare*, *T. aestivum*) on the resting spore populations and clubroot severity under both greenhouse and field conditions, and reached similar conclusions. That is, bait crops may be beneficial at moderate or low levels of resting spore inoculum; however, they had no effect on clubroot severity when field spore levels were high (1 × 10^6^ spores per gram of soil).

## 6 Conclusions and prospects

The increasing harm caused by *P. brassicae* to cruciferous crops has raised major concerns. By examining and condensing earlier reports, there is an enhanced comprehension of the biological characteristics and infestation mechanisms of *P. brassicae.* An increasing number of effectors associated with *P. brassicae* infestation have been identified, enhancing our understanding of the process by which *P. brassicae* infest their host. A variety of management strategies including planting resistant varieties, chemical control, biological control, farming practices and soil amendments have been shown to play an important role in controlling *P. brassicae* damage. Nonetheless, numerous issues remain to be addressed in the management of clubroot. First, the prevention of clubroot disease is essential. Accurate quantification of active spores is essential for predicting the severity of clubroot disease incidence. This places high demands on the accuracy and efficiency of quantification techniques. Establishing an accurate early prediction techniques system to assess disease risk is the focus and challenge of prevention work for clubroot. Second, the management of clubroot disease. There are very few registered chemicals available, and there is an urgent need to develop new, safe, efficient and economical agents. Biologists have consistently maintained great interest and enthusiasm in developing biocontrol agents. However, most biocontrol agents that have shown promising results in laboratory tests have yielded disappointing outcomes in practice practical applications. Nonetheless, researchers remain extremely passionate about trying to improve the stability of biocontrol agents. Most breeders believe that identifying resistance genes for use in breeding resistant varieties is the most reliable measure against *P. brassicae* infestation. However, resistant varieties have a risk of resistance break-down in the face of constantly evolving populations of *P. brassicae*. For this reason, scientists are constantly screening for new and effective resistance genes or polymerizing multiple resistance genes to develop resistant varieties to avoid risks.

The rapid development of bioinformatics and the emergence of new biotechnologies have accelerated the level of understanding of *P. brassicae*. On one hand, these technologies have deepened our understanding of the biological characteristics, pathogenic mechanisms, and catastrophic patterns of *P. brassicae* in a more comprehensive and scientific manner. On the other hand, they accelerate the analysis of the interaction between *P. brassicae* and host plants, as well as the interrelationships within the soil ecosystems in which they are located. Due to variations in soil acidity, cropping patterns, and field management across different regions, as well as potential differences in the physiological pathotypes of *P. brassicae*, developing a uniform management strategy to control this pathogen is challenging. Moreover, individual control measures are often inadequate for managing clubroot. With careful consideration to cost-effectiveness, the integrated application of multiple control measures, along with the development of region-specific clubroot management strategies, can significantly minimize the losses caused by *P. brassicae*. Specifically, it is the comprehensive use of ecological management, healthy cultivation, biological control, physical control, and the use of safe, efficient, and economical pesticides, and other measures to manage clubroot effectively. This approach aims to maximize social, economic, and ecological benefits (Figure 2). It is important to note that the actual effectiveness of such a management strategy that integrates multiple measures needs to be evaluated in detail and accurately across a large number of trials. In conclusion, it is essential to actively seek improvements in the current integrated control system for clubroot disease while minimizing reliance on chemical agents. This approach aims to prevent catastrophic outbreaks in the cruciferous plant production system caused by the *P. brassicae* and ultimately achieve sustainable management of clubroot disease.

**Figure 3:**
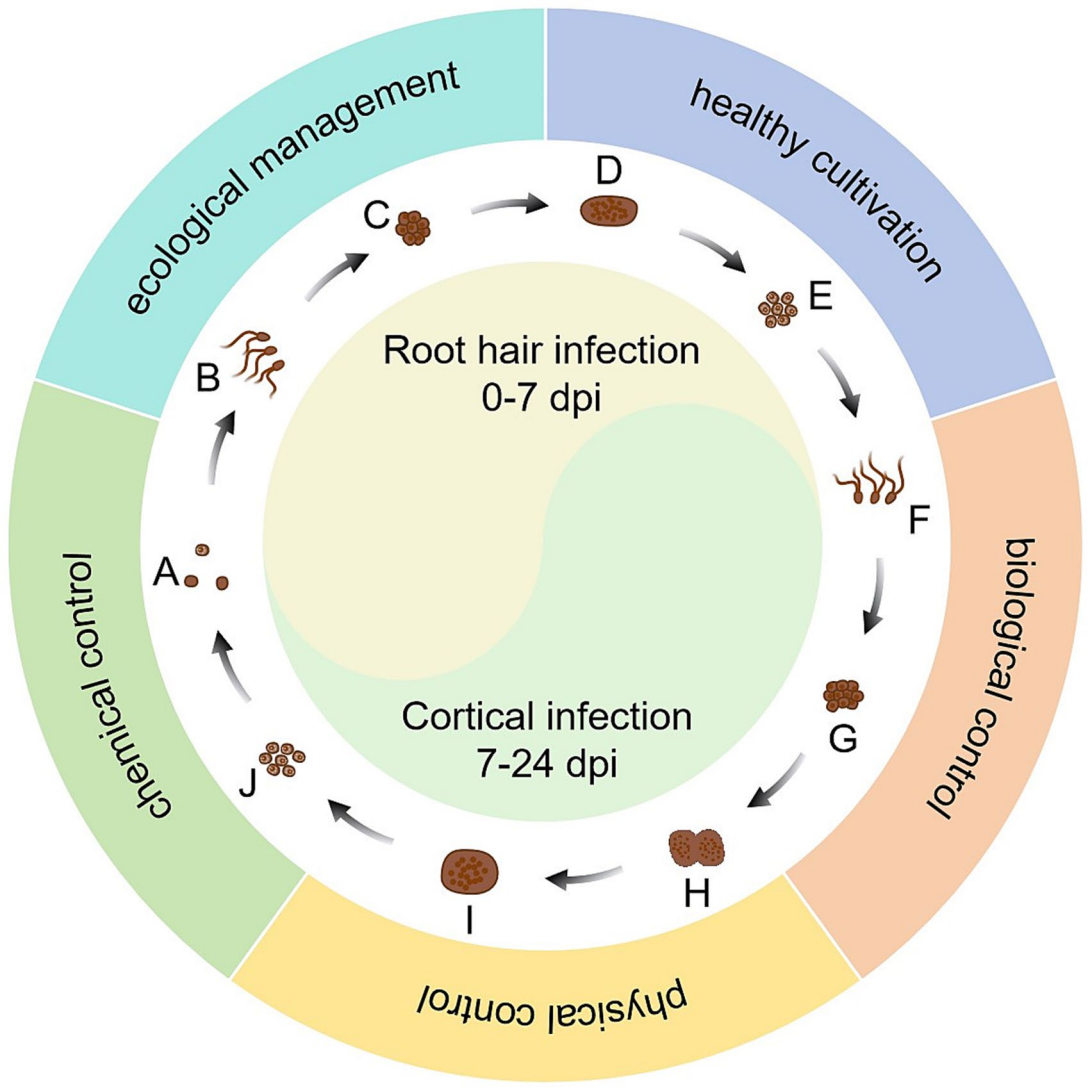
Integrated management of clubroot disease. (A) resting spores; (B) primary zoospores; (C) uinucleate primary plasmodium; (D) multinucleated zoosporangial plasmodium; (E) diploid uninucleate zygote; (F) secondary zoospores; (G) uinucleate secondary plasmodium; (H) multinucleated secondary plasmodium; (I) resting sporangial plasmodium; (J) resting spore formation.
